# Acetylated PLIN2 coordinates the innate immune system and oxylipin synthesis on lipid droplets to propagate inflammation

**DOI:** 10.21203/rs.3.rs-11406222/v1

**Published:** 2026-10-22

**Authors:** Charles Najt, Mahima Devarajan, Evan Kerr, Alex Churchill, Lauren Boatner, Samuel Gerrard, Brian Fess, Candace Guerrero, LeeAnn Higgins, Stacy Gelhaus, Douglas Mashek

## Abstract

Research has implicated lipid droplets (LDs) and their associated proteins in inflammation and the innate immune response. The key LD protein Perilipin 2 (PLIN2) is tightly correlated with lipid storage and inflammation, but little is known about the mechanisms underlying its proinflammatory effects. Here, we show that PLIN2 is required for the inflammatory response to mitogens and the recruitment of pro-inflammatory proteins to the surface of LDs. PLIN2 directly binds arachidonic acid and interacts with key oxylipin synthetic enzymes such as PLA2 and COX2 to promote LD-localized oxylipin synthesis and propagate inflammatory signaling. Moreover, LPS induces PLIN2 hyperacetylation, and acetylation-dead PLIN2 mutants lose their proinflammatory properties in the presence of an inflammatory stimulus, as if PLIN2 were ablated. Thus, acetylated PLIN2 forms a key metabolon on LDs that is essential for the response and amplification of proinflammatory signaling.

